# Nutrition and Pregnancy after Bariatric Surgery

**DOI:** 10.1155/2013/492060

**Published:** 2013-01-30

**Authors:** Lukasz Kaska, Jarek Kobiela, Anna Abacjew-Chmylko, Lukasz Chmylko, Magdalena Wojanowska-Pindel, Paulina Kobiela, Anna Walerzak, Wojciech Makarewicz, Monika Proczko-Markuszewska, Tomasz Stefaniak

**Affiliations:** ^1^Department of General, Endocrine and Transplant Surgery, Medical University of Gdansk, 17 Smoluchowskiego Street, 80-211 Gdańsk, Poland; ^2^Department of Gynecology, Gynecological Oncology and Endocrinological Gynecology, Medical University of Gdansk, 1A Kliniczna Street, 80-402 Gdańsk, Poland; ^3^Department of Neonatology, Medical University of Gdansk, 1A Kliniczna Street, 80-402 Gdańsk, Poland

## Abstract

Obesity is an escalating problem in all age groups and it is observed to be more common in females than males. About 25% of women meet the criteria of obesity and one-third of them are in the reproductive age. Because morbid obesity requiring surgical treatment is observed with increasing frequency, surgeons and gynecologists are undergoing new challenges. It is not only a matter of women's health and their quality of life but also proper development of the fetus, which should be a concern during bariatric treatment. Therefore complex perinatal care has to be provided for morbid obesity patients. The paper reviews pregnancy and fertility issues in bariatric surgery patients.

## 1. Incidence and Implications of Obesity

Obesity defined as body mass index (BMI) of 30 kg/m^2^ or greater is an emerging health problem in the modern world, above all in industrial countries. It is becoming the main cause of preventable morbidity and mortality. Public health research has identified obesity as the second leading cause of death in America, just after tobacco-related diseases [1]. 

It is estimated that about 27% of population in the United States is obese [2]. This problem is escalating in all age groups, with a greater occurrence in females. About 25% of women meet the obesity criteria and one-third of them are in the reproductive age [3]. Obese women are at higher risk of associated diseases. Some studies have demonstrated that BMI increase is associated with higher miscarriage rate and lower mature oocyte yield [4]. In other emphases that fetal evaluation during pregnancy is difficult. Furthermore, morbidly obese pregnant women are endangered by high risk pregnancy, delivery, and postpartum complications [2] (Table 1). 

The bariatric surgery is advisable only for carefully selected, morbidly obese patients when conservative methods of weight reduction were unsuccessful [5]. 

## 2. Pregnancy after Bariatric Surgery

 Pregnancy after surgical bariatric treatment appears to be a complex medical challenge [6]. It is necessary to inform women about supplementation and additional laboratory tests and possible negative influence of bariatric surgery on their future pregnancies. It is suggested that women after bariatric surgery who become pregnant need to be followed up by a group of specialists including a nutritionist, an educated nursing staff, an obstetrician, an endocrinologist, an internal medicine specialist, and a bariatric surgeon [7].

Considerations on pregnancy following bariatric surgery are dependent straightly on the performed type of procedure. Restrictive bariatric procedures provide weight loss through reducing stomach capacity, while malabsorptive operations diminish gastric volume and disrupt proper absorption of ingested food and nutrients. Each category of procedures has its own input on postoperative nutrition and the outcomes of pregnancy, especially that malabsorptive procedures are combined with higher risk of nutritional deficiency [3].

## 3. Preconception and Antepartum

As it has been previously mentioned, obese women often have problems with fertility, especially due to disturbances in ovulation (anovulation or oligoovulation) and increased risk of development of polycystic ovary syndrome (PCOS) [6]. After bariatric procedures, alongside with weight loss, their fertility performance is improved. When losing as little as 5% of initial body weight obese women with PCOS improve spontaneous ovulation rates and spontaneous pregnancy. It could be influenced by reducing insulin resistance, decreasing the level of androgens, reducing hirsutism, and stabilizing the level of sex hormones. Psychological factors like increased attractiveness after weight reduction have their impact as well [8, 9]. Radical weight loss may rarely have an adverse effect on fertility, as it may result in an temporary cessation of menstruations in the form of hypogonadotropic hypogonadism and only intensive hormonal therapy may result in recovering a normal cycle [8]. 

Currently it is highly recommended to delay pregnancy for 12–18 months after surgery [10], because of a rapid weight loss phase and its specific stressful influence on the organism. The starvation phase may be dangerous for both mother and fetus [11]. Although the malnutrition does not increase the risk of congenital defects, yet some studies reveal an increases risk of “small to gestational age” or restricted growth risk in infants among women with prior bariatric surgery and single studies noted increased risk of preterm birth or preterm rupture of membranes [12]. Therefore, in the first period after the procedure patients should consider the use of contraceptives [13]. The patients should be warned that oral contraceptives may not achieve adequate levels of their active substances in serum, because of altered absorption. Therefore, other routes of admissions should be advised, for example, transdermal or vaginal systems. 

## 4. Vomiting

In women who underwent bariatric surgery vomiting and nausea are frequent symptoms. All patients especially after restrictive operations may experience persistent vomiting when they do not thoroughly chew their food or eat too rapidly. Women in the first trimester of pregnancy frequently experience persistent vomiting due to high levels of B-hcG or decreased levels of progesterone. Vomiting as a result of pregnancy added to that from restriction can be much more difficult to handle [3, 14]. Pregnant women, who have undergone adjustable gastric banding and are experiencing hyperemesis, may have the band deflated or even opened to facilitate pouch emptying, for reducing the frequency of vomiting and for feeling temporary alleviation [14, 15]. Some authors recommend to deflate or open the band in pregnant women in all cases and to wait at least 6 months after birth to refill. Deflation of the band can be accomplished during early pregnancy by its access at the reservoir under the skin. In the case of other bariatric procedures it is necessary to perform upper GI endoscopy to study the gastrointestinal anastomosis. Stretching a narrowed opening is possible if needed, at that time. Only after pregnancy should CT or upper GI series be employed for diagnostics [9]. 

## 5. Nutritional and Microelements Deficiency

All patients after bariatric surgery have to take vitamins and microelements which prevent micronutrients' deficiency. Pregnant women have an increased requirement for microelements and vitamins, while in those who have undergone bariatric procedures it is even more important. Ideally, micronutrient deficiencies should be prevented or treated before a woman becomes pregnant [6]. Unfortunately, only 59% of women after bariatric surgery take multiple vitamin supplements for a long period of time [6, 16]. Remainder, in case of pregnancy, have higher risk of complications caused by deficiencies, especially as the deficiencies most frequently concern vitamin B_12_, folic acid, calcium, and iron [6]. Therefore, nutritional status during pregnancy and lactation may be a contributing factor in maternal and infant morbidity and mortality.

Common issue following bariatric surgery is calcium deficiency caused by its inadequate consumption or malabsorption. The bypass procedures lead frequently to its deficiency as a result of excluding the duodenum and proximal jejunum from calcium absorption [7]. It is recommended to increase the intake from 1000 mg of calcium citrate with 10 mcg vitamin D to 2000 mg of calcium citrate with vitamin D (50–150 mcg). The citrate form is optimal; it does not require acidic stomach environment to be broken down and absorbed [7]. Inadequate calcium intake may result in maternal bone loss, reduced breast milk calcium secretion, or inappropriate mineralization of fetus skeleton. To prevent restricted fetus growth, fetal biometry should be regularly monitored by ultrasound scan followups [7]. 

Another common pathology in women after bariatric surgery is iron deficiency anemia. The pathogenesis is multifactorial. It is partially related to decreased intake of adequate iron quantities. Additionally bariatric operated patients may develop achlorhydria which results in reduction of iron absorption. Bypassing duodenum and proximal jejunum eliminates the first and main site of the iron absorption [14, 17]. It is almost certain after malabsorptive operations. Patients, who have undergone restrictive operations, seldom experience iron deficiency and routine supplementation of it may not be required. However, to verify iron sufficiency it is desirable to control regularly serum levels of hemoglobin, iron, ferritin, and transferrin [7]. As prevention, iron should be taken in ferrous form not in a normal pregnancy dose of 30 mg daily but of 40–65 mg daily and the supplementation should be modified after laboratory tests [7].

Attention should be paid to the next anemia cause—vitamin B_12_ deficiency. Although being less common, almost each patient becomes regularly supplemented with cobalamin. Following bariatric surgery the absorption of the B_12_ may be impaired at different levels depending on the type of the procedure. Absence of acid environment following gastric bypass surgery, inadequate secretion of intrinsic factor (IF) and finally malabsorption result in the decrease of the amount of vitamin B_12_/IF absorption in the terminal ileum leading to vitamin B_12_ deficiency [15]. The low concentration of cobalamin may result in elevation of serum homocysteine. Hyperhomocysteinemia is directly related to early loss of pregnancy [18]. To avoid this complication homocysteine and vitamin B_12_ levels should be measured. Inappropriate level of vitamin B_12_ related with lower intake during pregnancy can result in neurobehavioral disorders in infant presenting symptoms of decreased ability to concentrate, depression, problems with abstract thought, and memory impairment and confusion. Severe vitamin B_12_ deficiency could also result in anemia in infants [18]. The recommended daily sublingual dose of cobalamin during pregnancy after bariatric surgery should be increased from 3 mcg to 10 mcg in easily absorbed crystalline form. This is generally sufficient to maintain adequate serum cobalamin levels which will normalize the level of homocysteine [7]. Occasionally when this therapy is not sufficient intramuscular injections are recommended in monthly dose 1000 mcg [15]. We suggest the modern sublingual substitution, which is more convenient for the patients.

During the last years a huge emphasis has been put on the folic acid and prevention of fetal neural tube defects (NTDs), which occur when the brain, skull, spinal cord, and spinal column do not develop properly within 4 weeks after conception. The most common NTDs are anencephaly, which causes stillbirth and death soon after delivery and spina bifida which may result in a wide range of physical disabilities including partial and total paralysis [14]. If maternal foliate stores are insufficient prior to a subsequent conception, the risk of adverse pregnancy outcome such as preterm delivery and birth defects in the following pregnancy increases. For this reason, it is recommended that all women of childbearing age who are capable of becoming pregnant should supplement the folic acid. Following the bariatric surgery rich in foliate food bypasses the duodenum or is not well absorbed by the patients so they have higher risk of folic acid deficiency and associated NTDs. Prenatal supplementation with vitamins containing 4 mg of folic acid prior to and during pregnancy is usually sufficient to maintain adequate serum levels to reduce the risk of neural tube defects; however, there are no strong proof for this [6, 19]. 

The next important consideration is vitamin A deficiency. It is reported that this deficiency exists in 10% of patients following gastric bypass [15]. The exclusion of the duodenum results in delayed mixing of dietary fat (with fat soluble vitamins) with pancreatic enzymes and bile salts which leads to malabsorption and deficiency as the consequence [15]. Vitamin A plays an important role in cell's reproduction, differentiation, and proliferation. The adequate level of the vitamin A is necessary especially in the second and third pregnancy trimesters for normal fetal lung development and maturation. Several studies have presented an increased risk of bronchopulmonary dysplasia (BPD) in preterm infants with insufficient vitamin A status [17]. Furthermore, vitamin A deficiency also impairs iron status, increases susceptibility to respiratory infections and diarrhea, and increases morbidity and mortality. It has been suggested that the vitamin A status of pregnant women can influence vitamin A stores in the fetus liver [20]. To avoid this complications plasma retinol levels have to be examined periodically and if necessary oral supplement therapy should be introduced, but without exceeding the dose of 5000 IU/d [6]. Nevertheless special attention is recommended by virtue of fact that excessive amount of the retinol is teratogenic for fetus [14]. Some studies recommend intake of *β*-carotene (nonteratogenic) instead of retinol [17, 21]. An additional benefit of vitamin A supplementation in pregnant women is increasing hemoglobin concentrations [20].

An additional concerning issue is vitamin K deficiency. There are no reports now on the number of pregnant women presenting this disturbance, but it is notable that vitamin K has already limited placental transfer during normal pregnancy. The excessive vomiting or fat malabsorbtion, affecting pregnant women after bariatric surgery, may lead to a higher risk of vitamin K-deficient bleeding disorders of the neonates of these mothers. Eerdekens et al. reported on five cases with severe intracranial bleeding and skeletal malformations similar to warfarin fetopathy (*Rhizomelic chondrodysplasia punctata*), which were caused by vitamin K deficiency of mothers following bariatric surgery. It is the first well-documented report relating to maternal vitamin K deficiency and following complications. To avoid severe malnutrition and vitamin K depletion, the authors of this paper recommend close followup with specialized team. However, there are no recommendations about supplementation of the vitamin K [22].

Not only vitamins' levels are disturbed in pregnancy or after bariatric procedures but also Zinc levels could decrease by about 30% during “normal” pregnancy, so it is imperative to have adequate supplies. It should be considered especially after malabsorptive bariatric operations. Low levels of zinc have been combined to premature deliveries, low birth weight, abnormal fetal development, and spina bifida. Cases of zinc deficiency during breastfeeding are accompanied by skin rashes or dermatitis as the main symptoms, often in combination with failure to thrive and irritability [22]. We suggest optimal dose of zinc which contained 15 mg a day.

Magnesium supplementation during pregnancy may reduce fetal growth retardation and preeclampsia and increase birth weight. Furthermore, it can help to prevent premature contractions by relaxing the muscles of the uterus. Studies demonstrated that magnesium levels were lower in women who have had a premature labor [23, 24]. During pregnancy requirement for magnesium rises two times; however, supplementation is obligatory at the dose of 200–1000 mg daily if states of deficiency occur or when symptoms of deficiency appear [25].

Iodide deficiency is revealed in over half of all pregnant women, similarly as in the overall population. The requirement for iodide during pregnancy rises twice especially during the first trimester; therefore WHO recommends its daily intake at the level of 250 mcg; however, only 150 mcg should be supplemented while the rest absorbed during nutrition. The advised supplementation in the preconception period is 50 mcg [25]. Unfortunately, there are no recommendations for the pregnant women after bariatric treatment. Due to the threat of malnutrition we suggest the intake of 250 mcg of iodide. 

Antioxidant nutrient status in pregnancy is also a potentially interesting issue. Oxidative stress caused by free radicals has been implicated in many studies on preeclampsia etiology. It is a dangerous condition in pregnancy which possibly leads to poor fetal growth and premature birth. It could result in serious complications for the woman involving liver, kidneys, brain, or blood clotting system. Antioxidants, such as vitamin C, vitamin E, selenium, and lycopene, could neutralize free radicals. The current evidence does not support the use of antioxidants to reduce the risk of preeclampsia or other pregnancy complications, but clinical trials are still in progress [26]. Moreover, selenium deficiency may be a factor in some early-pregnancy miscarriages. However, while adequate selenium is necessary for normal development of a fetus, some evidence suggest that too much selenium may be a reason of nervous-system damage [27]. Until the results of a randomized trial are not published, it is not recommended that women should supplement selenium during pregnancy.

In summary, in order to prevent complications in pregnancy after bariatric surgery such as micronutrient deficiencies and associated disorders, patients must be precisely informed about postoperative necessity of supplementation intake. In addition periodical serum examination has to be conducted and if necessary the dose of vitamins or microelements should be changed for certain individuals. It is worth mentioning that recent studies on pregnancy after gastric-bypass surgery have proved no adverse perinatal outcomes associated with micronutrient deficiencies [12, 28, 29].

## 6. Gestational Diabetes and Hypertension

It is important to emphasize that the incidence of gestational diabetes and hypertension is lower in pregnancies after bariatric procedures [15] and it should be described as an undeniable benefit for the woman and fetus. Standard testing for gestational diabetes in women following bariatric surgery is problematic. In pregnant women the glucose tolerance test could cause “dumping syndrome” with nausea, abdominal cramps, diarrhea, and heart palpitation [3]. In this special group of patients the fasting blood sugar test or continuous glucose monitoring for several days should be recommended [3, 14].

## 7. Weight Gain

Weight gaining during pregnancy following bariatric surgery can be diverse. Women who have undergone adjustable gastric banding and sleeve gastrectomy present the highest variation in weight gain. It is reported that some of them lose weight during pregnancy, while other gain more than it was recommended. However, it is observed that women who delay pregnancy for at least 2 years are most likely to have a restricted or normal pregnancy weight gain [7]. Because of potential abnormal fetus growth, regular check examination must be performed, including fetus ultrasounds and clinical examination [7]. In addition those women should undergo ultrasound scans more frequently than the general population (every 4 to 6 weeks starting from the 24th week of gestation) to monitor fetus' growth [30]. The most recent information about outcomes after obesity surgery suggests that guidelines for weight gain during pregnancy after bariatric treatment should be revised. Commonly, normal-weight women with BMI of 19.8–26 are recommended to gain 11.5–16 kg, while those in the “high range” (BMI of 26.1–29) should have a “recommended target weight gain of at least 7 kg”—according to the Institute of Medicine of the National Academy of Sciences.

## 8. Intrapartum 

A normal and uncomplicated course of labor is expected in the postbariatric surgery pregnant women. It is reported that women who are stabilized following bariatric surgery and achieve nutritional balance usually experience less morbidity and mortality during pregnancy [3]. Significant weight loss decreases the risk of intrapartum complications such as preeclampsia, large gestational age infant, operative delivery, and surgical wound infections [7] which are frequent in obese women pregnancies.

However, there are some bariatric surgery related complications which can appear during perinatal period and lead to severe outcomes including fetus and/or maternal death. Pregnant women following bariatric procedures may develop abdominal hernias, gallstones, gastrointestinal hemorrhage, internal herniation of the bowel all due to changes in nutrient absorption, changes in metabolism, and organ displacement as the uterus enlarges [3, 14]. It is reported that there are three time periods when obstruction is more probable to occur: when the uterus becomes an abdominal organ, during the labor, and during the postpartum period when the uterus involutes [28]. These complications are rare; nevertheless it is very important to inform pregnant women about the urgency of these conditions and ask to report symptoms like severe pain, nausea, vomiting, fever, and flu-like symptoms [3].

## 9. Postpartum

As it is recommended by the WHO to breastfeed for at least 6 months, mothers after bariatric surgery also should be encouraged to breastfeed their newborn children. It is obvious and undeniable that maternal nutrition has an impact on the quality of their milk. It has to be concerned that the fetus may develop malnutrition, especially when mother has underwent malabsorptive bariatric procedure. It is essential to maintain micronutrients supplementation also after delivery and during breastfeeding, to ensure appropriate vitamins and minerals intake to neonate and to prevent vitamin B deficiency, which can indicate severe complications including failure in thriving, megaloblastic anemia, and development delays [3, 14]. It was reported that after adequate vitamin B supplementation improvements in growth, development and reversal of anemia are observed [7]. Entire period of breastfeeding should be under special medical care the same as pregnancy. We perform medical controls every 3 months in our pregnant and breastfeeding women (Medical Universities in Gdansk and Freiburg).

## 10. Summary

As obesity is a growing problem in modern societies, the number of women in the childbearing age after bariatric surgery will be increasing, as well as the number of pregnant women after surgical obesity treatment. Recent studies have documented that to prevent the adverse medical outcomes during and after pregnancy following bariatric procedures, the suitable medical care must be provided. In addition, the cooperation between the medical staff and well-informed pregnant women is important and indispensable. Some studies have suggested that the laparoscopic adjustable gastric banding (LAGB) or sleeve gastrectomy are more suitable for young women than Roux-en-Y gastric bypass or Biliopancreatic Diversion but definitive studies comparing these two procedures have to be conducted. After malabsorptive bariatric surgery all pregnant patients should be precisely examined with supplementation and if necessary supplemented. Patients have to be informed about avoiding pregnancy in the first year after bariatric surgery and about the insufficiency of oral contraception. The period of pregnancy and breastfeeding should be correlated with the laboratory tests' controls in each trimester and later every three months with the adequate supplement modification. Currently there are no recommendations for pregnant women following bariatric surgery [28]. Further research is needed before drawing definitive conclusions.  Table 2 indicates major issues involving the care of these patients. 

Bariatric surgery is the most intensely developing area of surgical practice today, so we expect facing new long-term effects with reproductive issues as one of the most important ones to be assessed.

## Figures and Tables

**Table 1:** Maternal risks associated with obesity.

Period	Increased risk
Antenata	Increased incidence of miscarriage Gestational diabetes mellitus Hypertension and preeclampsia Fetal anomalies Venous thromboembolism

Intrapartum	Failure to progress the labor Shoulder dystocia Fetal monitoring problems The need of emergency cesarean section Surgical complications

Postpartum	Wound infection Venous thromboembolism Postnatal depression

**Table 2:** Major recommendations for women following bariatric surgery.

Reliable contraception for 12–18 months after surgery	

During pregnancy one standard prenatal vitamin daily, which may include or should be supplemented with the following: (i) 1000–2000 mg of calcium citrate with vitamin D addition (50–150 mcg) daily (ii) 40–65 mg of ferrous iron daily (iii) 350 mcg of cobalamin sublingual daily or 1000 mcg of cobalamin intramuscular per month (iv) 4 mg of folic acid daily (v) 15 mg of zinc daily	

Laboratory tests' controls including the levels of iron, hemoglobin, ferritin, transferrin, calcium, homocysteine, cobalamin, and retinol	

Regularly ultrasound scans followup which evaluated the fetus growth and mineralization of the skeleton	

Close followup of weight changes during pregnancy and postpartum	
